# Open Surgical Placement of T9-T12 Dorsal Root Ganglion Stimulators With Titanium Plate Anchor Fixation in a Patient With Recalcitrant Post-herpetic Neuralgia

**DOI:** 10.7759/cureus.15040

**Published:** 2021-05-15

**Authors:** Ryan Johnson, Jason M Seibly

**Affiliations:** 1 Neurosurgery, Carle BroMenn Medical Center, Normal, USA; 2 Neurosurgery, Central Illinois Neuroscience Foundation, Bloomington, USA

**Keywords:** dorsal root ganglion, neuromodulation, post-herpetic neuralgia, laminectomy

## Abstract

Dorsal root ganglion stimulators (DRGS) have been used to treat patients with neuropathic pain due to multiple etiologies. Typically, DRGS are inserted percutaneously with fluoroscopic guidance epidurally into the neuroforamina over a pathologic dorsal root ganglion. In patients with unfavorable anatomy due to extensive surgical scarring, an open surgical approach has been described in the literature for DRGS placement. We document an alternative open surgical approach for DRGS placement in a patient with recalcitrant post-herpetic neuralgia.

## Introduction

Neuropathic pain results from damage to components of the central nervous system and often has a delayed presentation after healing from the primary nerve injury is complete [1, 2]. The pathophysiologic mechanism of neuropathic pain is still not completely understood. Pharmacotherapies targeted at non-neuropathic sources of pain have been shown to provide sub-optimal neuropathic pain relief.

Modulation of neuronal hyperexcitability has since become an area of surgical pursuit in this patient population. Dorsal root ganglion (DRG) electrical stimulation has been shown to reduce neuronal excitability and has emerged as a neuromodulation technique that is non-inferior to dorsal column spinal cord stimulation (SCS) in selective pain etiologies [1,3]. Placement of a DRG electrode is performed percutaneously with fluoroscopic guidance to ensure placement of the electrode within the intervertebral foramen. However, in patients with a history of multiple spine surgeries, epidural scarring may impede the accurate placement of the electrode. Additionally, the task of anchoring the leads in situations that do not permit strain relief loops to prevent lead migration has not been extensively documented. Open surgical placement of DRG electrodes has been documented in two patients with successful placement in both cases [4]. We detail a technical note reporting our experience with the open surgical placement of a DRG stimulator in a 65-year-old female with refractory post-herpetic neuralgia.

## Case presentation

**Figure 1:**
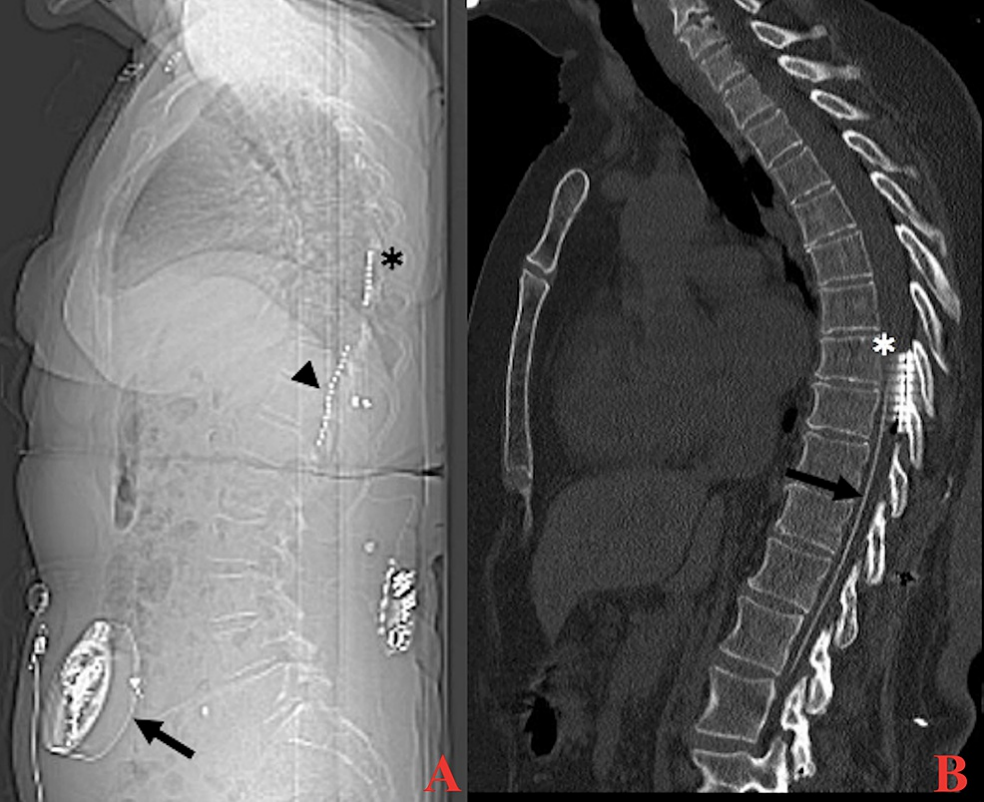
Lateral x-ray (A) and sagittal computed tomography of the thoracic spine (B) demonstrating the intrathecal analgesia pump reservoir and intra-dural catheter (black arrow), single percutaneous electrode (arrowhead), and paddle electrode (asterix).

The patient is a 65-year-old female with a complicated history of post-herpetic neuralgia with neuropathic pain involving the left T9-T12 dermatomes that resulted in her being placed on disability. Her prior surgical history included an intrathecal analgesia pump, percutaneous and paddle dorsal column stimulators, all of which failed to provide durable pain relief (Figures 1A, 1B). To better delineate which dermatome was causative to her symptoms, left-sided selective nerve root blocks were performed at T9, T10, T11, and T12. Individually, these did not provide significant neuropathic pain relief, but the combination of all four selective nerve root blocks resulted in more than 50% reduction of her neuropathic pain. At this time, trial percutaneous placement of left T9-T12 dorsal root ganglion stimulators (DRGS) was attempted and failed secondary to significant epidural scarring precluding access of the electrodes to the neuroforamen. After a multidisciplinary conversation, open surgical placement of a left-sided T9-T12 DRGS was recommended to the patient. Written informed consent detailing the risks and benefits of surgical intervention was obtained.

The patient was brought to the operating room, underwent general anesthesia induction, and was placed in the prone position on a Spinal Jackson table. C-arm fluoroscopy was utilized to localize the incision over the T7-T12 spinal regions. Standard thoracic spine exposure with subperiosteal dissection technique was performed for exposure of the T7-T12 laminas. T8 and T9 laminectomies were performed. A significant amount of epidural scar was encountered at those levels and required careful dissection. This allowed removal of the solitary electrode and paddle lead from prior SCS surgeries. Bilateral thoracic laminectomies were then performed in standard fashion using Kerrison rongeurs and the microsurgical pneumatic drill from T10 to T12. The spinous process of each level was removed in order to facilitate the placement of the DRG electrode into the foramen by allowing a more favorable medial-to-lateral insertion angle. On the right side of the thoracic lamina from T10 to T12, a thin ridge of lamina adjacent to the facet joint was intentionally not removed to help with anchoring the leads later in the operation. Careful removal of the ligamentum flava both centrally and in the lateral recesses permitted exposure of the underlying thoracic dura. Partial left-sided T9 to T12 foraminotomies were performed by undercutting the bone overlying the intervertebral foramen with Kerrison rongeurs. Extensive epidural scarring was encountered and required careful dissection to create room for the DRG electrode insertion.

**Figure 2:**
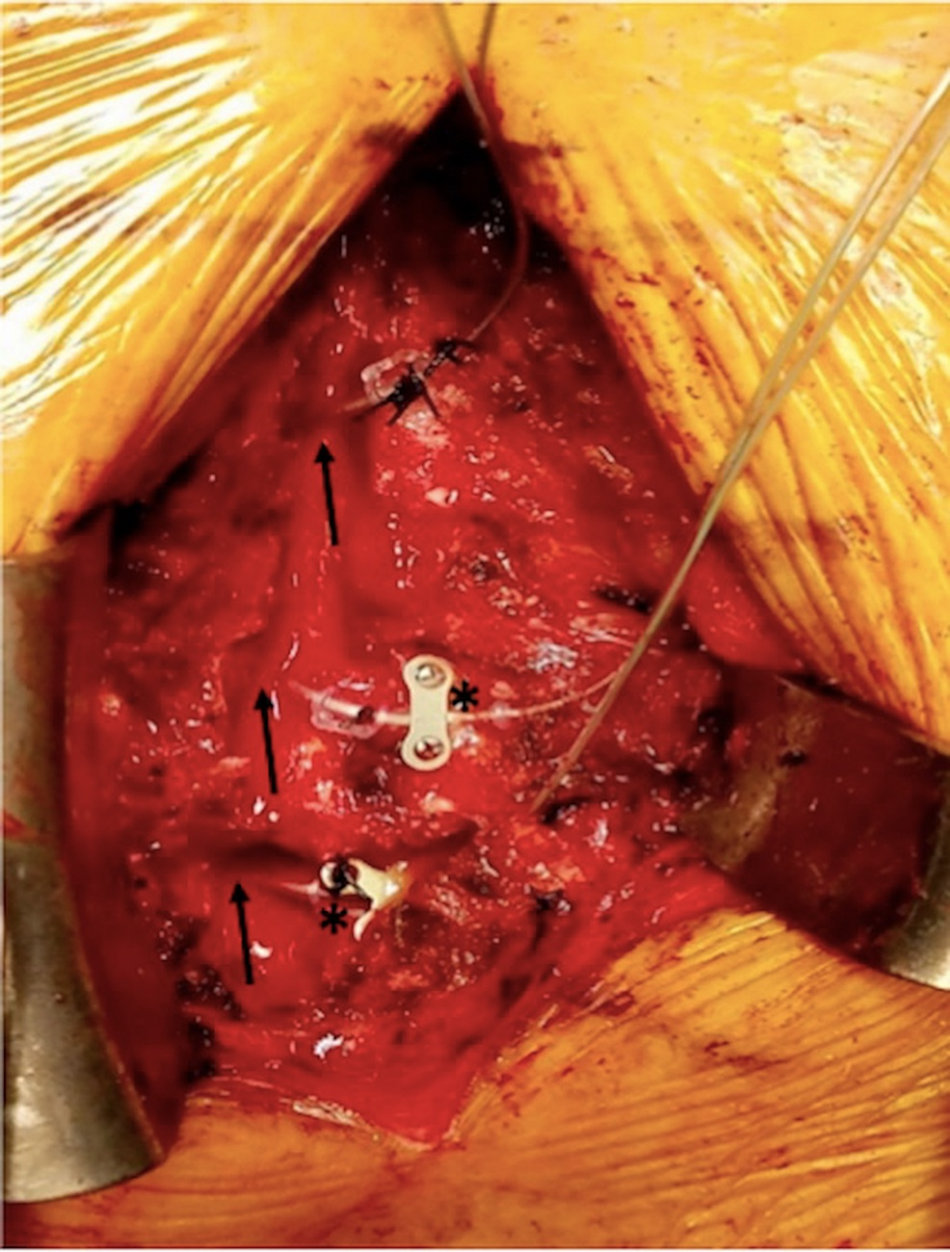
Intraoperative view demonstrating titanium cranial fixation plates (asterix) anchoring DRGS electrodes to the residual right-sided thoracic lamina. The T10-T12 DRG electrodes can be seen entering the intervertebral foramina (black arrows).

**Figure 3:**
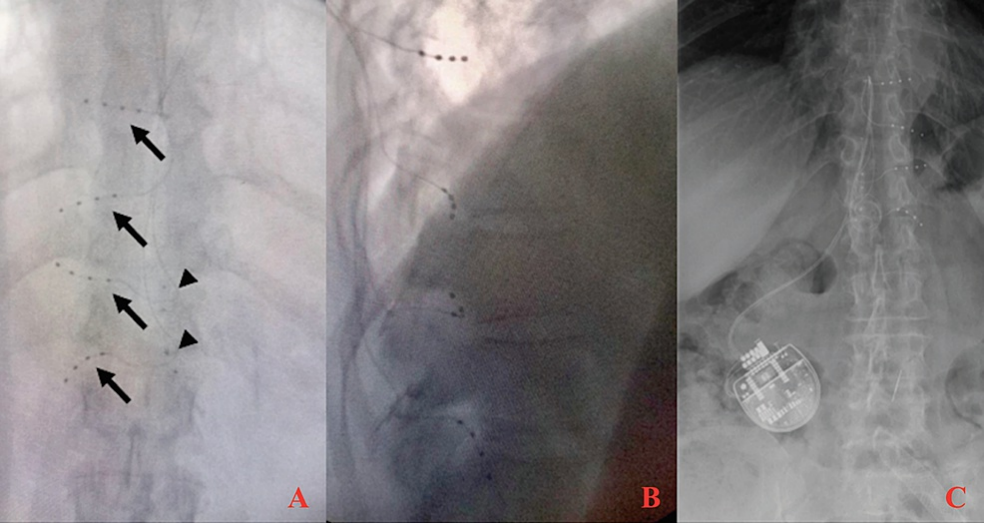
Anteroposterior (A) and lateral (B) fluoroscopic views demonstrating DRG electrodes (black arrows) exiting the left T9-T12 intervertebral foramina with titanium plates (arrowheads) anchoring the T11 and T12 electrodes. Additionally, a six-month follow-up abdominal x-ray (C) demonstrating a stable position of the DRG electrodes.

An Abbott ProclaimTM DRG Neurostimulation (Abbott Neuromodulation, Plano, TX, USA) linear electrode with four contacts was inserted into the intervertebral foramen at T9 to T12 using bayonet forceps under direct observation. Anteroposterior and lateral C-arm fluoroscopy was then utilized to confirm the location of the electrodes in the respective intervertebral foramen with the contacts providing coverage in the area corresponding to the dorsal root ganglion inferomedial and lateral the pedicle. Anchoring of T9 and T10 stimulator leads were accomplished with plastic anchors that were secured to the paraspinal scar tissue and fascia. This was felt to be sufficient to prevent electrode migration and subsequent withdrawal from the dorsal root ganglion. At T11 and T12, there was an absence of paraspinal scar tissue, and it was felt the fascia alone would not provide sufficient strength to prevent electrode migration. To solve this, the plastic anchoring device was placed over the electrode, followed by a small titanium cranial fixation plate placed over the lead, which then was secured using four millimeter titanium screws to the residual right-sided lamina at T11 and T12 (Figure 2). This proved to provide a stable anchor for each electrode without any evidence of lead impingement. A tunneling trocar was then used to tunnel subcutaneously to the internal pulse generator site previously determined from prior surgeries. The trochar was removed and the four leads were passed through the tunneling sheath and connected to an Abbott ProclaimTM DRG Neurostimulation internal pulse generator. Impedances were assessed and found to be within normal limits. C-arm fluoroscopy was used once again for final radiographs, which confirmed the proper location of each electrode on AP and lateral views. The wound was irrigated with a copious amount of antibiotic irrigation and closure proceeded in standard multi-layer fashion. Follow-up radiographs demonstrated a stable position of the leads six months post-operatively (Figures 3A-3C). Post-operatively, the patient had transient improvement in her neuropathic pain; but, unfortunately, the procedure did not provide long-term relief and the patient remains on disability.

## Discussion

DRG neurostimulation has been demonstrated in a prospective, multi-center, and randomized controlled trial to be superior to conventional SCS for patients with neuropathic pain secondary to complex region syndrome (CRPS) type II [3]. Since that landmark trial, DRG neurostimulation has been used successfully for treatment of multiple etiologies of neuropathic pain [1,3]. Surgical technique, in normal anatomic conditions, involves a percutaneous approach under fluoroscopic guidance to insert an electrode into the intervertebral foramen and the need for epidural strain relief loops to prevent lead migration. In the absence of normal anatomy due to prior surgical procedures, DRG stimulator implantation has been considered contraindicated.

Open surgical placement of DRG electrodes has been performed on two patients in Germany for radicular pain and CRPS [4]. Anchoring of the leads in both patients was accomplished with epidural strain relief loops and fibrin glue. Our patient illustrates another technical approach to open surgical placement of DRG electrodes in a situation of unfavorable anatomy for epidural strain relief loops to secure the leads. The titanium cranial fixation plates adequately secured the lead without impingement or disruption of impedances.

Even with proper placement of the electrodes, the patient continued to have significant neuropathic pain. The weakness of our management approach is the absence of trial stimulation with DRGS prior to permanent placement to document efficacy. Trial stimulation was attempted unsuccessfully due to epidural scarring precluding access to the neuroforamen in our patient. The patient did understand the risks of the procedure, including a realistic expectation that she may have minimal pain relief and was willing to try a more aggressive surgical approach. This case illustrates the difficulty in treating recalcitrant neuropathic pain secondary to post-herpetic neuralgia and mandates a multidisciplinary approach to ensure proper patient selection and treatment.

## Conclusions

Open surgical placement of a DRGS is a safe option for patients with post-herpetic neuralgia that have anatomic restrictions limiting placement percutaneously. From a technical standpoint, additional surgical case reports are needed to determine a standard open surgical approach. This approach has the distinct advantage of direct visualization of the intervertebral foramen to allow accurate placement of the electrode. The disadvantages to this approach are inherent to the actual invasiveness of the procedure requiring open surgical placement of each electrode. While in the thoracic spine, hemilaminectomies do not result in iatrogenic spinal instability, the risk of instability development would be greater in the cervical and lumbar spine; therefore, careful consideration of the patient's anatomy would be required in these locations to avoid the need for surgical fixation.
